# Pharmacotherapy of the spleen and stomach in Li Shizhen’s Bencao Gangmu (Compendium of materia medica, 1596): a text-statistical analysis of 994 entries and a bridge to modern pharmacology

**DOI:** 10.3389/fphar.2026.1889778

**Published:** 2026-09-16

**Authors:** Wan Sun

**Affiliations:** 1 Hubei University of Chinese Medicine, Wuhan, Hubei, China; 2 Hubei Shizhen Laboratory, Wuhan, Hubei, China

**Keywords:** Bencao Gangmu, digestive system, ethnopharmacology, Li Shizhen, medicinal plants, spleen–stomach disorders, text mining, traditional Chinese medicine

## Abstract

**Background:**

Bencao Gangmu (本草纲目, 1596), Li Shizhen’s Compendium of Materia Medica, is among the most comprehensive pre-modern compilations of East Asian materia medica, yet the full-text distribution of its content on the spleen–stomach (*pi-wei* 脾胃) system, the digestive functional axis of Chinese medicine, has never been systematically quantified or mapped onto modern pharmacological evidence.

**Aim of the study:**

To construct a reproducible text-statistical inventory of all spleen- and stomach-related entries in Bencao Gangmu; to characterize the resulting pharmacopoeia by plant identity, taste–nature (*qi-wei*), channel tropism (*gui-jing*), declared function, indication and preparation form; and to relate the most frequently recorded botanical drugs to current pharmacological evidence on gastrointestinal disease.

**Materials and methods:**

The Jinling (1596) edition was searched in full text for *pi* (脾), *wei* (胃) and *pi-wei* (脾胃). Candidate hits were adjudicated by two reviewers against predefined inclusion criteria (Cohen’s κ = 0.84) and coded into a relational dataset (drug, taste–nature, channel tropism, function, indication, preparation). Plant identities were authenticated against World Flora Online and the Chinese Pharmacopoeia (2025) and reported under the ConPhyMP framework.

**Results:**

Of 994 retained entries, 703 specified a concrete drug or therapy. The most frequent botanical drugs were *Atractylodes macrocephala* Koidz. (*Baizhu*, n = 26), *Zingiber officinale* Roscoe (*Shengjiang*, n = 25) and *Atractylodes lancea* (Thunb.) DC. (*Cangzhu*, n = 20). Sweet (n = 16), warm (n = 15) and slightly-cold (n = 14) taste–natures dominated; channel tropism converged on the spleen (32%) and the spleen–stomach pair (26%); the leading functions were spleen-tonifying (n = 46) and qi-replenishing (n = 41); the leading indication was *fan-wei* (food regurgitation, n = 108). Decoction accounted for 79% of preparation records and medicinal porridge (*yao-zhou*) for 11%.

**Conclusion:**

Bencao Gangmu codifies a balanced spleen–stomach pharmacotherapy prioritizing sweet–warm tonification counterweighted by a deliberate cooling–draining arm. The leading botanical drugs converge on phytochemical leads, including atractylenolides, gingerols/shogaols, atractylodin/β-eudesmol, berberine and processed-*Pinellia* alkaloids, with documented effects on gastrointestinal motility, mucosal protection, gut-microbiota modulation and anti-inflammatory activity, providing a reproducible bridge between classical Chinese materia medica and contemporary ethnopharmacology.

## 1 Introduction

Traditional Chinese medicine (TCM) attributes a central role in human physiology to the so-called “spleen–stomach” system (*pi-wei* 脾胃), conceived not as the two organs of modern anatomy but as the functional axis responsible for the intake, transformation and distribution of food essence (Maciocia, 2015; Scheid et al., 2015). Pathology of this axis is invoked to explain a wide spectrum of conditions whose clinical phenotypes overlap with functional dyspepsia, gastro-oesophageal reflux, irritable bowel syndrome, malnutrition and chronic gastritis in the biomedical nosology (Wu et al., 2020; Ji et al., 2021). Because much of the primary terminology of this field has no exact biomedical equivalent, all TCM technical terms in this paper are given at first occurrence with *pinyin*, Chinese characters and an English rendering that follows the WHO international standard terminologies on traditional medicine (WHO Regional Office for the Western Pacific, 2007; WHO, 2022), cross-checked against Wiseman and Feng (1998); a consolidated glossary with working definitions is provided in Table 1 for readers without a TCM background.

**TABLE 1:** Glossary of TCM technical terms used in this study.

Term (pinyin, Chinese)	English rendering (WHO/Wiseman)	Working definition
*pi* 脾	Spleen	TCM functional system governing transformation and transportation of food essence and generation of qi and blood; not identical with the anatomical spleen
*wei 胃*	Stomach	Functional system that receives and “ripens” ingested food before passing it on for transformation
*pi-wei 脾胃*	Spleen–stomach	The paired functional axis of digestion; the “root of the postnatal constitution”
*qi 气*	qi	Vital energy/functional activity of the body and its organ systems
*qi-wei 气味*	Nature and flavour (taste–nature)	Combined classification of a drug by thermal nature (cold–cool–warm–hot) and flavour (sweet, bitter, pungent, sour, salty, bland)
*gui-jing 归经*	Channel entry (channel tropism)	The organ channel(s) to which a drug’s action is held to be directed
*fu-zheng 扶正*	Supporting the right (body-reinforcing)	Treatment principle of strengthening the body’s own physiological capacities
*qu-xie 祛邪*	Eliminating pathogenic factors	Treatment principle of expelling pathological agents (dampness, heat, cold, food stagnation, phlegm)
*ben-xu-biao-shi 本虚标实*	Root deficiency with branch excess	Pattern in which an underlying deficiency coexists with superimposed excess manifestations
*fan-wei 反胃*	Stomach reflux (food regurgitation)	Vomiting of undigested food hours after intake (“eat in the morning, vomit in the evening”); overlaps phenotypically with gastroparesis-like motility disorders
*yao-shi-tong-yuan 药食同源*	Medicine and food homology	Doctrine that medicines and foods share a common origin; basis of dietary therapy
*yao-zhou 药粥*	Medicinal porridge	Congee-based dietary-medicinal preparation
*bu-xiao-bing-yong 补消并用*	Simultaneous tonifying and dispersing	Strategy combining reinforcement of deficiency with elimination of excess in one formula

English renderings follow the WHO international standard terminologies (WHO Regional Office for the Western Pacific, 2007; WHO, 2022), cross-checked against Wiseman and Feng (1998). Definitions are working glosses for non-TCM readers, not claims of biomedical equivalence.

The conceptual history of spleen–stomach medicine long predates its doctrinal systematization, and Bencao Gangmu stands at the confluence of several distinct lineages. The Huangdi Neijing (Yellow Emperor’s Inner Classic, ca. 2nd century BCE) already designated the spleen and stomach as the “root of the postnatal constitution” (*hou-tian zhi ben* 后天之本), the source from which qi and blood are generated after birth. Practical pharmacotherapy of the middle burner is equally ancient: Zhang Zhongjing’s Shanghan Lun and Jin Gui Yao Lue (ca. 200 CE) codified formulas such as Li Zhong Wan (理中丸, Regulate-the-Middle Pill) for cold deficiency of the middle and Zhi-Zhu Tang (枳术汤) combining *Atractylodes* and immature bitter orange—evidence that the core drug repertoire preceded the mature theory by a millennium. The theory itself crystallized in the Jin–Yuan period: Zhang Yuansu of the Yishui school introduced the systematic attribution of drugs to organ channels (the direct ancestor of the channel-tropism data analyzed in Section 3.4), and his student Li Dongyuan’s Pi Wei Lun (Treatise on the Spleen and Stomach, 1249) elevated the spleen–stomach to the pivot of internal medicine with the dictum that “internal damage to the spleen and stomach gives rise to the hundred diseases.” A further counterpoint was added by Zhu Danxi, whose yin-nourishing doctrine warned against injuring stomach yin through excessive warm-dry treatment. Li Shizhen (1518–1593), writing three centuries later, explicitly synthesized these lineages, and the entries analyzed in this study therefore encode not a single school but the accumulated, internally balanced consensus of Han formula practice, Yishui organ-channel pharmacology and Danxi yin-protection.

Bencao Gangmu (本草纲目, Compendium of Materia Medica), completed by Li Shizhen and printed in 1596, is widely regarded as the most comprehensive pre-modern compilation of East-Asian materia medica, listing 1,892 drugs across 52 juan (volumes) and incorporating roughly 11,000 formulas (Nappi, 2009; Métailié, 2015). It remains an actively mined source of candidate botanical drugs for modern pharmacological and text-mining research (Zhang et al., 2022; Chung et al., 2024), with a 2026 methodological review of annotation and indexing approaches for such texts underscoring the continued relevance of this line of work (Liu et al., 2026). For the spleen–stomach domain in particular, Bencao Gangmu is uniquely valuable for three reasons: (i) it integrates the materia-medica tradition with extensive cross-references to the Pi Wei Lun lineage; (ii) it preserves a large number of dietary-medicinal (*yao-shi-tong-yuan* 药食同源, “medicine and food share a common origin”) preparations not found in earlier compilations; and (iii) it records preparation-specific details (processing, decoction, porridge) that are essential for ethnopharmacological reproducibility (Unschuld, 2018; Bian, 2020).

Three considerations make the pharmacotherapy of the spleen and stomach a domain particularly worthy of systematic study. First, the clinical burden is large: the functional gastrointestinal disorders onto which *pi-wei* pathology phenotypically maps, including functional dyspepsia, gastroparesis, irritable bowel syndrome, affect a substantial share of the world population, and their pharmacological options remain limited (Camilleri et al., 2018; Wu et al., 2020), so that a prioritized inventory of historically vetted botanical candidates has direct discovery value. Second, spleen–stomach theory is arguably the most widely applied doctrinal framework in contemporary TCM digestive practice, underlying the majority of modern gastrointestinal prescriptions in China (Ji et al., 2021); quantifying its most authoritative historical source therefore documents the textual basis of a living clinical tradition. Third, among classical sources Bencao Gangmu is the largest single repository of this tradition, yet its spleen–stomach content has never been exhaustively inventoried; a reproducible quantification thus fills a defined gap at the intersection of medical history and ethnopharmacology.

Several recent studies have applied data-mining to classical Chinese medical texts, either to identify candidate botanical drugs for specific indications—diabetic nephropathy (Zhang et al., 2014), rheumatoid arthritis (Xia et al., 2020), migraine (Zhang et al., 2022) and memory disorders (May et al., 2013) — or to generate innovative formulas *de novo* (Chung et al., 2024), the latter group extending this approach in 2025 to antimicrobial lead discovery through combined text-mining and molecular computation (Chung et al., 2025). At the pharmacopoeia scale, quantitative ethnobotanical analysis has recently been applied to the single botanical drugs of the Ayurvedic Pharmacopoeia of India (Yao et al., 2023), and quantitative work on Graeco-Roman materia medica has shown that chemosensory qualities predict recorded therapeutic use (Leonti et al., 2024), demonstrating that historical pharmacopoeias are tractable objects of rigorous quantitative analysis. However, these studies have generally drawn on broad classical-text corpora (e.g., Zhong Hua Yi Dian) rather than performing an exhaustive, single-source analysis of a foundational pharmacopoeia, and their disease focus has been narrowly biomedical. To our knowledge, no published work has provided a comprehensive, reproducible text-statistical inventory of the spleen–stomach pharmacotherapy embedded in Bencao Gangmu, nor mapped this inventory onto contemporary phytochemical and pharmacological evidence.

The present study addresses this gap: the complete Jinling (1596) edition of Bencao Gangmu was searched for every entry containing *pi* (脾), *wei* (胃) or *pi-wei* (脾胃), yielding 994 adjudicated substantive medical mentions. This study characterizes the resulting pharmacopoeia along six dimensions—plant identity, taste–nature, channel tropism, declared function, indication and preparation form—and then discusses five of the most frequently used botanical drugs in the light of modern phytochemical and pharmacological evidence relevant to gastrointestinal pathophysiology. The work is intended as a hypothesis-generating ethnopharmacological resource and as a reproducible methodological template for further text-statistical studies of historical materia medica.

## 2 Materials and methods

### 2.1 Source text and editorial choices

The reference text was the Jinling (Nanjing) edition of Bencao Gangmu, printed by Hu Chenglong in the 24th year of the Wanli reign (AD 1596). This is the earliest extant complete edition and is the standard scholarly base for textual research (Métailié, 2015). The full-text Jinling edition held by the digital archive of the National Library of China was used as the searchable corpus; ambiguous passages were cross-checked against the People’s Health Publishing House critical edition (Liu, 2004) and the punctuated edition by Liu (2014). No translation was introduced at the entry-adjudication stage; all judgments were made on the classical Chinese source.

### 2.2 Search strategy and entry adjudication

The corpus was searched for three character strings: *pi* (脾), *wei* (胃), and the bigram *pi-wei* (脾胃). Each candidate hit was extracted with its surrounding clause (typically 30–80 characters) and adjudicated against the following inclusion criteria: (i) a substantive reference to spleen and/or stomach physiology, pathology, treatment principle, drug action or indication; and (ii) sufficient context to assign at least one of the six analytical dimensions (drug, taste–nature, channel tropism, declared function, indication, preparation). Exclusion criteria were (i) purely literal or geographic mention; (ii) exact duplication; and (iii) mention only in cross-references without independent content.

The corpus was coded by the author (W.S.) and independently adjudicated by two external reviewers (S.H. and P.W.). Inter-rater agreement between the two adjudicators on a 200-entry random subset, calculated as Cohen’s κ, was 0.84 (95% CI 0.79–0.89). Residual disagreements were resolved by consensus discussion among the three. A total of 994 entries were retained, of which 703 specified a concrete drug or therapy and constitute the analytical sample for botanical-drug-level analyses (Sections 3.2–3.6).

### 2.3 Plant authentication and ConPhyMP reporting

In line with the Consensus-based reporting guidelines for Phytochemical Characterisation of Medicinal Plant extracts (ConPhyMP) for ethnopharmacological reporting (Heinrich et al., 2022), every plant taxon discussed in the present manuscript has been authenticated under its currently accepted binomial. Names follow World Flora Online (https://www.worldfloraonline.org/) and Plants of the World Online (POWO; https://powo.science.kew.org/), with family attribution according to APG IV. For each plant this study additionally provides the family, the Pinyin name, the part used (organ) and the cross-reference to the Chinese Pharmacopoeia, 2025 edition (ChP 2025; Chinese Pharmacopoeia Commission, 2025), where applicable. Authenticated identities for the botanical drugs discussed in the text are summarized in Table 2; the complete list for all botanical drugs appearing in Figure 1 is given in Supplementary Table S1.

**TABLE 2:** Authenticated identities of the principal medicinal plants discussed in this study.

Accepted binomial (WFO/POWO)	Family (APG IV)	Pinyin (Chinese)	Part used	n	ChP 2025 monograph
*Atractylodes macrocephala* Koidz	Asteraceae	Baizhu 白术	Rhizome	26	Atractylodis Macrocephalae Rhizoma
*Zingiber officinale* Roscoe	Zingiberaceae	Shengjiang 生姜 (fresh)/Ganjiang 干姜 (dried)	Rhizome	25/5	Zingiberis Rhizoma Recens/Zingiberis Rhizoma
*Atractylodes lancea* (Thunb.) DC.	Asteraceae	Cangzhu 苍术	Rhizome	20	Atractylodis Rhizoma
*Pinellia ternata* (Thunb.) Makino	Araceae	Banxia 半夏	Tuber	14	Pinelliae Rhizoma
*Syzygium aromaticum* (L.) Merr. and L.M.Perry	Myrtaceae	Dingxiang 丁香	Flower bud	12	Caryophylli Flos
*Aconitum carmichaelii* Debx	Ranunculaceae	Fuzi 附子 (processed)	Lateral root (processed)	11	Aconiti Lateralis Radix Praeparata
*Magnolia officinalis* Rehder and E.H.Wilson	Magnoliaceae	Houpo 厚朴	Bark	11	Magnoliae officinalis cortex
*Tetradium ruticarpum* (A.Juss.) T.G.Hartley (syn. *Evodia rutaecarpa*)	Rutaceae	Wuzhuyu 吴茱萸	Near-ripe fruit	11	Euodiae Fructus
*Coptis chinensis* Franch	Ranunculaceae	Huanglian 黄连	Rhizome	11	Coptidis Rhizoma
*Aucklandia costus* Falc	Asteraceae	Muxiang 木香	Root	10	Aucklandiae Radix
*Scutellaria baicalensis* Georgi	Lamiaceae	Huangqin 黄芩	Root	9	Scutellariae Radix
*Piper nigrum* L	Piperaceae	Hujiao 胡椒	Fruit	7	Piperis Fructus
*Citrus × aurantium* L	Rutaceae	Zhishi 枳实	Immature fruit	5	Aurantii Fructus immaturus
*Cimicifuga heracleifolia* Kom. (= *Actaea heracleifolia* (Kom.) J.Compton)	Ranunculaceae	Shengma 升麻	Rhizome	4	Cimicifugae Rhizoma
*Alpinia officinarum* Hance	Zingiberaceae	Gaoliangjiang 高良姜	Rhizome	—	Alpiniae officinarum Rhizoma
*Dioscorea polystachya* Turcz	Dioscoreaceae	Shanyao 山药	Rhizome	—	Dioscoreae Rhizoma
*Nelumbo nucifera* Gaertn	Nelumbonaceae	Lianzi 莲子	Seed	—	Nelumbinis semen

Binomials follow World Flora Online/Plants of the World Online; families follow APG IV; pharmacopoeial monograph names follow the Chinese Pharmacopoeia, 2025 edition (Chinese Pharmacopoeia Commission, 2025). n = number of mentions among the 703 botanical-drug-specifying entries (as in Figure 1); “—” marks botanical drugs discussed in the text that fall outside the fifteen most frequent (full data in Supplementary Data S1). Complete identities for all botanical drugs are also listed in Supplementary Table S1.

**FIGURE 1:**
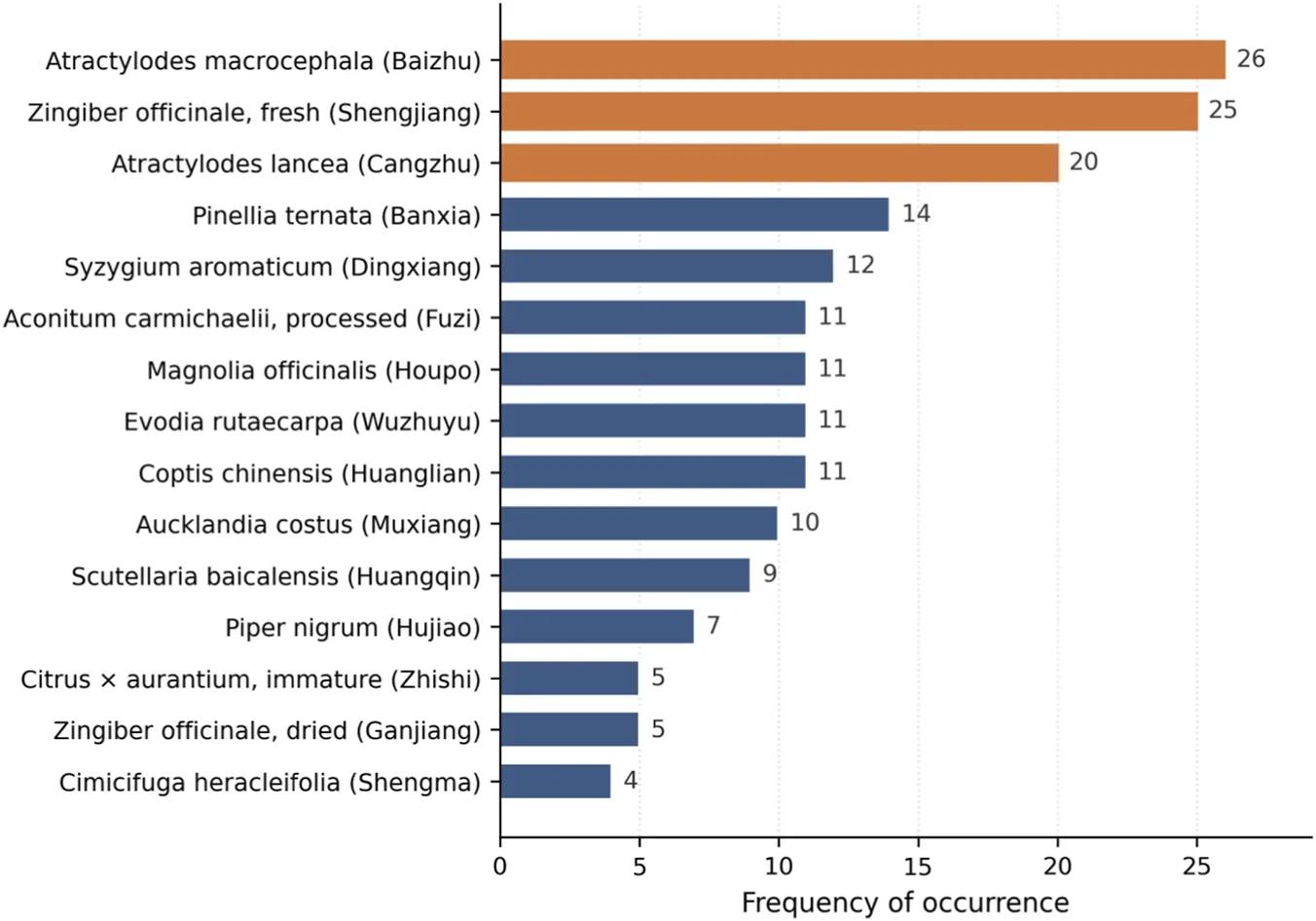
The fifteen most frequently mentioned botanical drugs in the spleen- and stomach-related entries of Bencao Gangmu. Frequencies are based on the 703 botanical-drug-specifying entries. Plant names follow World Flora Online; Pinyin in parentheses. The top three botanical drugs are highlighted (orange). Authenticated identities and family attributions for all botanical drugs are provided in Supplementary Table S1.

Because the present work is an analysis of a historical text rather than a study of laboratory preparations, no plant extract was newly produced for this manuscript and no *in-vitro* or *in-vivo* experiment was performed. Where modern pharmacological evidence is invoked in the Discussion, this study cites published primary studies that meet ConPhyMP standards for botanical characterization.

### 2.4 Data structuring and statistical analysis

The overall analytical design—exhaustive extraction of a closed pharmacopoeial corpus followed by categorical frequency analysis—follows the quantitative ethnobotanical approach recently applied to the single botanical drugs of the Ayurvedic Pharmacopoeia of India (Yao et al., 2023). Each retained entry was coded along the following fields: entry ID, juan and chapter, drug or therapy mentioned, declared taste–nature, declared channel tropism, declared function tag, indication, preparation form, and verbatim citation. Taste–nature labels followed the standard 23-term TCM lexicon, channel tropism followed the 12-meridian framework, function tags followed the ChP 2025 classification, and indication labels were normalized against the WHO International Standard Terminologies on Traditional Medicine in the Western Pacific Region (WHO Regional Office for the Western Pacific, 2007) and cross-checked against the updated WHO international standard terminologies on traditional Chinese medicine (WHO, 2022) and the Wiseman lexicon (Wiseman and Feng, 1998) to keep translated terms consistent for non-Chinese readers (Table 1). The twelve function tags analyzed in Section 3.5 (Figure 2) are not an *ad hoc* scheme: they instantiate the standard drug-action (gongxiao 功效) categories of Chinese materia medica pharmacology, formalized in the ChP 2025 monograph headers, each rendering a recurrent classical descriptor already present, in varying wording, in the Bencao Gangmu entries themselves—for example, jian-pi 健脾 for “spleen-tonifying” or zhi-ou 止呕 (also jiang-ni zhi-ou 降逆止呕, “descending counterflow qi to arrest vomiting”) for “anti-emetic”. The English gloss “anti-emetic” therefore renders a long-standing traditional action category rather than a modern biomedical import; its modern-sounding form reflects the translation convention adopted throughout this paper (WHO Regional Office for the Western Pacific, 2007; WHO, 2022), not an anachronistic reclassification of the source material.

**FIGURE 2:**
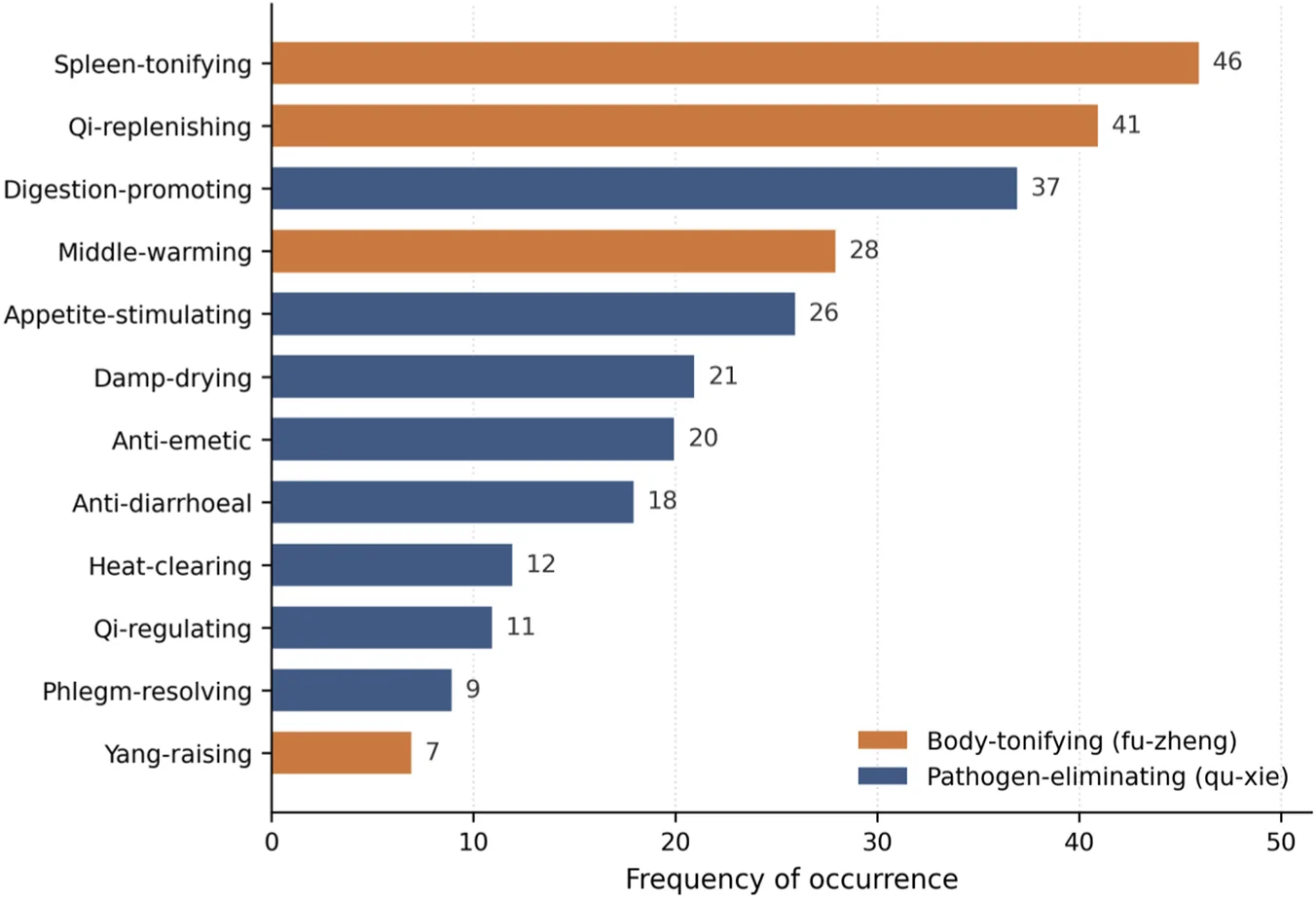
Frequency of declared functions for spleen–stomach drugs in Bencao Gangmu. Frequencies count function-tag occurrences across the 703-entry analytical sample (one drug may have multiple tags). Function labels follow the standard ChP 2025 drug-action classification; see Section 2.4 for the classical basis of individual labels (e.g., “anti-emetic” renders zhi-ou 止呕). Orange bars denote body-tonifying (fu-zheng) functions; blue bars denote pathogen-eliminating (qu-xie) functions. The two leading functions are tonifying, but the cumulative volume of pathogen-eliminating functions is greater, illustrating the body-vs-pathogen balance characteristic of Li Shizhen’s pharmacotherapy.

Frequency and proportional analyses were performed in Python 3.10 (pandas 2.0, NumPy 1.26) with figures produced in Matplotlib 3.7. For each categorical dimension this paper reports raw frequencies and the corresponding proportions of the analytical sample. Because the source is a single closed corpus and the analysis is descriptive, no inferential testing was applied. The complete coded dataset is provided as Supplementary Material (Supplementary Data S1, .csv format) and is additionally deposited at https://github.com/arielsun1990/Supplementary-Data to enable reproduction and re-analysis.

### 2.5 Literature search for the modern-pharmacology bridge

To contextualize the most frequently mentioned botanical drugs against current evidence (Section 4.2), this study performed a structured literature search in PubMed and Web of Science (Core Collection) using the authenticated Latin binomial in combination with the terms (“gastrointestinal” OR “gastric” OR “intestinal” OR “digestive” OR “motility” OR “mucosa” OR “microbiota”) covering 2010–2026. Only studies reporting an *in-vivo* or *in-vitro* experiment with adequately characterized plant material (in line with ConPhyMP) or rigorous clinical evidence were considered. The bridge discussion is illustrative, not exhaustive.

## 3 Results

### 3.1 Overall distribution of spleen- and stomach-related entries

Of the 994 retained entries, 496 (49.9%) referred to the spleen alone, 272 (27.4%) to the stomach alone, 224 (22.5%) to the spleen and stomach jointly, and 2 (0.2%) listed the two organs in apposition. The predominance of “spleen-only” entries reflects the TCM conception of the spleen as the master organ of transformation, qi-blood generation, fluid metabolism and blood-holding (Maciocia, 2015), and is consistent with Li Shizhen’s documented endorsement of the Yishui school (Zhang Yuansu, Li Dongyuan) view of the spleen as the post-natal root.

The 703 entries that specified a concrete drug or therapy constitute the analytical sample for Sections 3.2–3.6.

### 3.2 High-frequency botanical drugs and combinatorial tendencies

The fifteen most frequently mentioned botanical drugs are shown in Figure 1, and the authenticated identities of the principal botanical drugs discussed below are summarized in Table 2. Three botanical drugs dominate: *Atractylodes macrocephala* Koidz. (Asteraceae; Baizhu; n = 26), *Zingiber officinale* Roscoe—fresh rhizome (Zingiberaceae; Shengjiang; n = 25), and *Atractylodes lancea* (Thunb.) DC. (Asteraceae; Cangzhu; n = 20). Together with *Pinellia ternata* (Thunb.) Makino (Araceae; Banxia; n = 14), *Syzygium aromaticum* (L.) Merr. and L.M.Perry (Myrtaceae; Dingxiang; n = 12) and *Coptis chinensis* Franch. (Ranunculaceae; Huanglian; n = 11), these six botanical drugs anchor a broader clinical strategy that, across the top-fifteen list of Figure 1, resolves into four functional axes: spleen-tonifying/damp-drying (the *Atractylodes* pair), middle-warming (*Zingiber*, *Syzygium*, *Tetradium ruticarpum* (A.Juss.) T.G.Hartley (syn. *Evodia rutaecarpa*), *Aconitum carmichaelii* Debx. processed), heat-clearing/damp-drying (*Coptis chinensis*, *Scutellaria baicalensis* Georgi, *Pinellia ternata*) and qi-regulating (*Aucklandia costus* Falc., *Magnolia officinalis* Rehder and E.H.Wilson, *Citrus* × *aurantium* L. immature).

*Atractylodes macrocephala* merits particular attention because Li Shizhen explicitly incorporates Zhang Yuansu’s nine canonical uses for this drug (“Baizhu jiu yong”). Three pairs of these uses appear contradictory at first sight—warming the middle yet clearing stomach heat, generating fluids yet drying dampness, stopping perspiration yet promoting urination—but resolve once the underlying logic is recognized: heat in the spleen–stomach is conceived as the secondary product of trapped dampness, so that drying dampness removes the source of heat without the direct cold-bitter approach that would itself injure the spleen yang (Zhu et al., 2018). Li Shizhen also records detailed processing variants, such as earth-stir-fried (*tu-chao* 土炒) for diarrhoea, bran-fried (*fu-chao* 麸炒) for general spleen tonification, raw for diuresis, which significantly modify the *in-vivo* pharmacological profile of atractylenolides (Wang et al., 2026).

The most consistent pairing recorded is *Atractylodes macrocephala* + *Citrus* × *aurantium* immature fruit (枳术丸, Zhi-Zhu Wan), a tonify-and-reduce dyad that became the structural template of countless later digestive formulas. The dyad itself is far older than its doctrinal elaboration: it originates in Zhang Zhongjing’s decoction Zhi-Zhu Tang (Jin Gui Yao Lue, ca. 200 CE), was converted to pill form and re-rationalized by Zhang Yuansu, and was propagated by Li Dongyuan as the model “tonify the root while reducing the branch” formula, which is a textbook illustration of how an ancient empirical combination was progressively re-theorized as spleen–stomach doctrine matured (see Introduction). Li Shizhen further records targeted modifications: adding *Coptis chinensis* for heat, *Zingiber officinale* (dried) for cold, and *Pinellia ternata* for phlegm—documented evidence that Zhi-Zhu Wan was already a mature scaffold formula in the 16th century.

### 3.3 Distribution of taste–nature (qi-wei)

The taste–nature distribution of the botanical drugs (Figure 3) is dominated by sweet (n = 16), warm (n = 15) and slightly-cold (n = 14), followed by pungent-warm (n = 11), cold (n = 8), sweet-warm (n = 6), hot (n = 5) and bitter (n = 5). Sweet-tonifying and warm-supporting flavours together account for the largest share, consistent with the canonical maxim that “the spleen prefers warmth and dryness while the stomach prefers cool moisture” (脾喜温燥, 胃喜润凉; Pi Wei Lun, juan 1).

**FIGURE 3:**
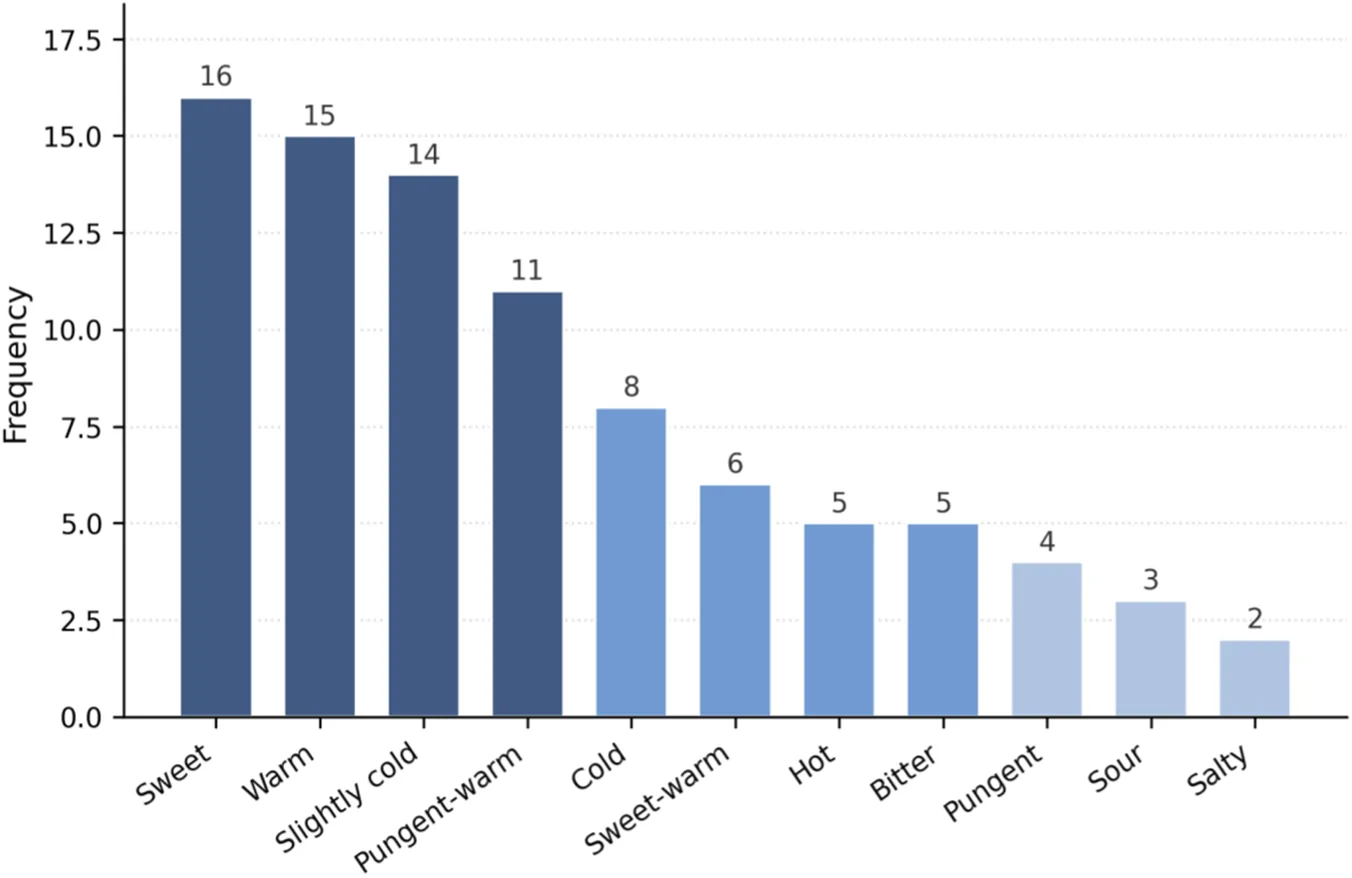
Distribution of declared taste–nature (qi-wei) for spleen–stomach drugs in Bencao Gangmu. Frequencies count drug-by-entry occurrences in the 703-entry analytical sample. Bar colour reflects relative magnitude. Sweet and warm dominate, with a substantial cool-cold counterweight that reflects the dual physiology of the spleen (prefers warm-dry) and the stomach (prefers cool-moist).

This dual taste–nature profile is the chemical–thermodynamic signature of the wider therapeutic logic: tonifying-warming botanical drugs (*Atractylodes*, *Zingiber*, *Aconitum*) are systematically balanced by cooling-bitter botanical drugs (*Coptis*, *Scutellaria*), so that neither the spleen yang nor the stomach yin is unilaterally compromised. The relatively high frequency of slightly-cold drugs (n = 14) — usually clearing-and-harmonizing rather than purgative—corresponds in particular to the stomach’s physiological intolerance of unopposed warming. The premise that taste classification encodes genuine pharmacological information is supported by recent quantitative evidence outside the Chinese tradition: in a phylogenetically controlled analysis of 700 botanical drugs of Graeco-Roman materia medica, perceived taste and flavour qualities significantly predicted recorded therapeutic uses (Leonti et al., 2024), suggesting that the taste-based *qi-wei* system analyzed here reflects a cross-cultural, chemosensation-grounded logic of drug selection rather than arbitrary convention.

### 3.4 Channel tropism (gui-jing)

Channel tropism (Figure 4) is sharply concentrated on the spleen (31.9%) and the spleen–stomach pair (25.5%), which together account for 57.4% of all tropism assignments. The remainder is distributed across taiyin (8.5%), yangming (6.4%), spleen–lung (6.4%), spleen–kidney (4.3%), stomach alone (4.3%) and the shaoyang qi-aspect (4.3%), with a residual 8.5% spread across minor tropism assignments not individually named (see “Other” in Figure 4).

**FIGURE 4:**
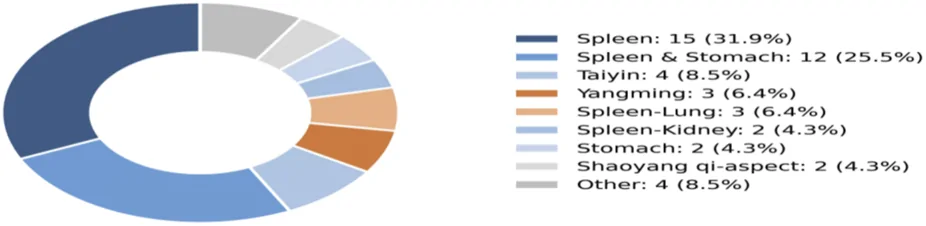
Channel tropism (gui-jing) of spleen–stomach drugs in Bencao Gangmu. Proportions calculated from the 703-entry analytical sample. The spleen and the spleen + stomach pair together account for over half of all tropism assignments, illustrating Li Shizhen’s preference for targeted rather than broad-spectrum action. Spleen–lung and spleen–kidney co-tropism reflect the integrated physiology of post-natal qi generation.

The dominance of spleen-targeted tropism reflects a deliberate strategy of “targeted delivery” — Li Shizhen consistently prefers drugs whose action is held to be locally directed rather than systemically diffused. This channel-attribution system itself is a direct inheritance from Zhang Yuansu’s Yishui school (see Introduction), which Li Shizhen adopted and extended. At the same time, secondary tropism toward the lung and kidney is not accidental: lung qi depends on spleen earth (“earth generates metal” 土生金), while kidney essence is replenished by spleen-derived nutriment, and the spleen–lung and spleen–kidney co-tropisms (*Dioscorea polystachya* Turcz. — Shanyao; *Nelumbo nucifera* Gaertn. seed—Lianzi) capture exactly this integrated physiology.

### 3.5 Declared functions

The most frequently declared functions (Figure 2) are spleen-tonifying (n = 46), qi-replenishing (n = 41), digestion-promoting (n = 37), middle-warming (n = 28), appetite-stimulating (n = 26), damp-drying (n = 21), anti-emetic (n = 20) and anti-diarrhoeal (n = 18). When the categories are grouped into body-tonifying (*fu-zheng* 扶正) and pathogen-eliminating (*qu-xie* 祛邪) functions, tonifying functions account for the two top slots but the cumulative volume of pathogen-eliminating functions (digestion-promoting, damp-drying, anti-emetic, anti-diarrhoeal, heat-clearing, qi-regulating, phlegm-resolving) is in fact larger. (The classical basis of these function labels, including “anti-emetic”, is described in Section 2.4.)

This distribution mirrors the canonical view of spleen–stomach disease as “deficiency at the root, excess at the branch” (本虚标实): tonification addresses the underlying weakness while a broader, more granular set of pathogen-eliminating functions deals with the symptomatic surface—exactly the structure of the Zhi-Zhu Wan template documented in Section 3.2.

### 3.6 Main indications

The most frequent indications (Figure 5) are dominated by *fan-wei* (反胃, regurgitation/vomiting of ingested food; n = 108), followed by abdominal distension (n = 41), vomiting (n = 24), spleen–stomach deficiency (n = 22), spleen deficiency (n = 20), diarrhoea (n = 18), cold-stomach syndrome (n = 14), stomach-heat syndrome (n = 11), dysentery (n = 10) and hiccup (n = 8). The dominance of *fan-wei* is striking: the symptom complex—eating in the morning and vomiting in the evening, eating in the evening and vomiting in the morning—overlaps phenotypically with upper-gastrointestinal motility disorders, including gastroparesis and several forms of functional dyspepsia (Camilleri et al., 2018), though the historical and biomedical nosologies are not mutually translatable.

**FIGURE 5:**
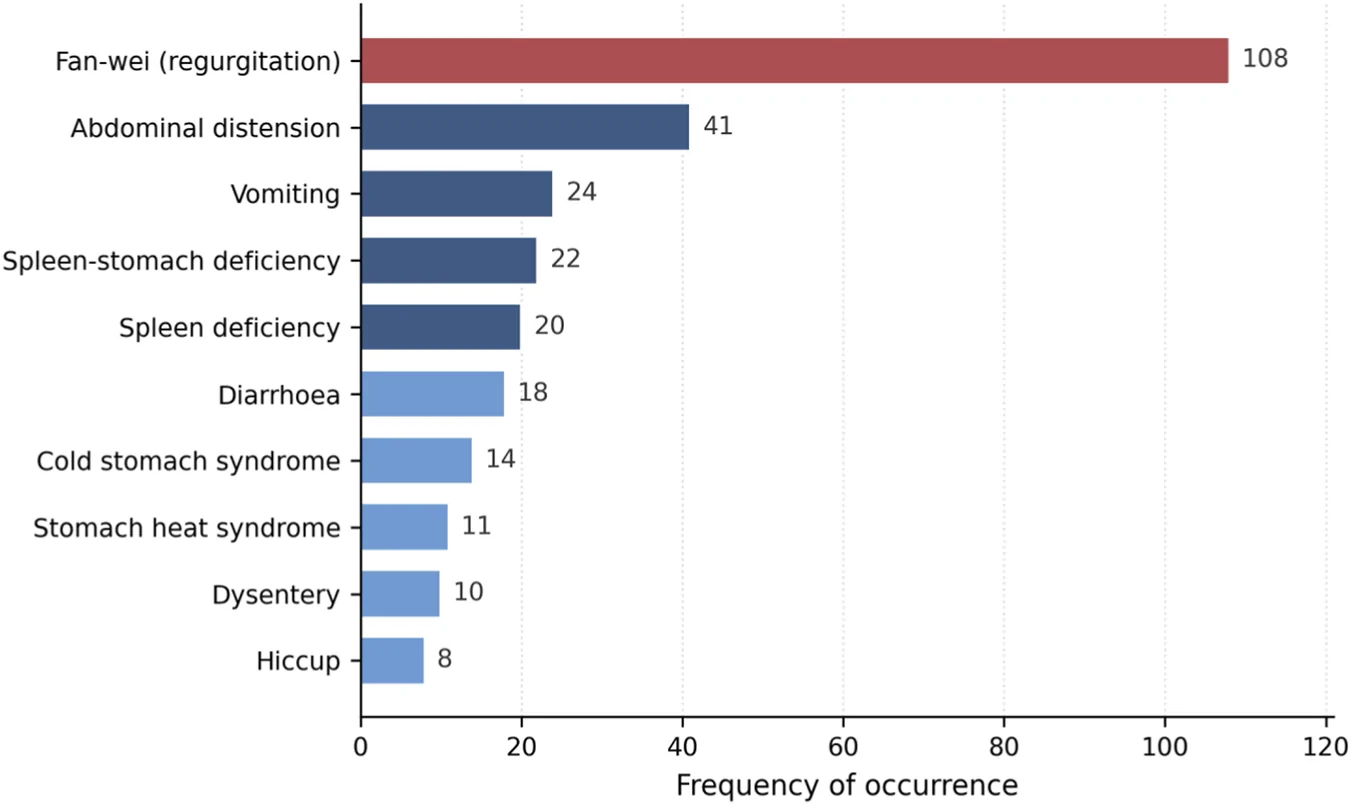
Frequency of main indications targeted by the spleen–stomach drugs of Bencao Gangmu. Fan-wei (regurgitation/food vomiting) dominates the indication profile (highlighted red) and corresponds phenotypically to disorders of upper-gastrointestinal motility. Categories were normalized against the WHO International Standard Terminologies on Traditional Medicine in the Western Pacific Region (WHO Regional Office for the Western Pacific, 2007).

The indication profile preserves a clean cold–heat differential (cold-stomach n = 14 vs. stomach-heat n = 11), confirming that Li Shizhen explicitly distinguished the cold and hot variants of the same anatomical complaint and chose drugs accordingly (e.g., *Zingiber officinale* dried rhizome and *Alpinia officinarum* Hance for the cold pole; *Coptis chinensis* and Gypsum fibrosum for the hot pole).

### 3.7 Preparation forms

Preparation forms (Figure 6) are dominated by oral decoction (*jian-fu* 煎服, n = 53; plus decoctions of a single botanical drug, n = 14; together 79%), followed by medicinal porridge (*yao-zhou* 药粥, n = 9; 11%), pill (*wan* 丸, n = 4; 5%) and chewable (*jue-fu* 嚼服, n = 1; 1%).

**FIGURE 6:**
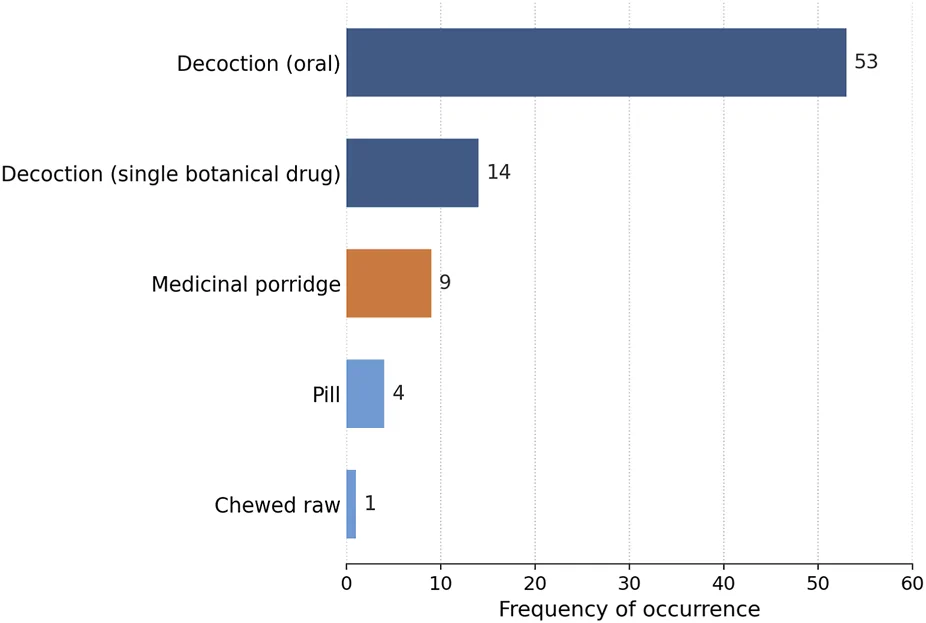
Distribution of preparation forms for spleen–stomach drugs in Bencao Gangmu. Frequencies count preparation-form occurrences across the 703-entry analytical sample. Decoction dominates (79%), but medicinal porridge (yao-zhou) is a non-trivial 11%, illustrating the integration of dietary therapy (yao-shi-tong-yuan) into spleen–stomach pharmacotherapy.

Two features of this distribution merit comment. First, decoction dominates because of its acknowledged advantages for spleen–stomach pathology: high bioavailability of polar metabolites, ease of dose titration, and rapid feedback in clinical follow-up. Second, the 11% share of medicinal porridge is non-trivial: it captures the integration of dietary therapy (*yao-shi-tong-yuan*) into spleen–stomach pharmacotherapy. Examples include *Dioscorea polystachya* porridge for spleen-deficient diarrhoea and *Nelumbo nucifera* seed porridge for combined spleen–kidney depletion. These preparations remain part of contemporary Chinese clinical nutrition and supply a plausible dietary route for bioactive metabolites whose pharmacokinetics in concentrated decoction may be poorly tolerated by frail patients (Zhang et al., 2019).

## 4 Discussion

### 4.1 A coherent five-feature signature of Li Shizhen’s spleen–stomach pharmacotherapy

Across five of the six analytical dimensions coded in this study—taste–nature, channel tropism, declared function, indication and preparation form; plant identity is treated separately in Sections 3.2 and 4.2 — a coherent five-feature signature emerges. First, the dominant taste–nature combination is sweet + warm, balanced by a smaller but deliberate cool-clearing arm; this reflects the dual physiology of the spleen (warm-dry preference) and the stomach (cool-moist preference). Second, channel tropism is sharply targeted at the spleen and spleen–stomach pair, with secondary co-tropism to the lung and kidney that mirrors the integrated post-natal physiology. Third, the functional profile combines a tonifying core (spleen-tonifying, qi-replenishing) with a larger pathogen-eliminating periphery (digestion-promoting, damp-drying, anti-emetic, anti-diarrhoeal), producing the canonical “deficiency at root, excess at branch” structure. Fourth, indications focus on motility-related syndromes—*fan-wei*, abdominal distension, vomiting, diarrhoea—with a clean cold–heat differential preserved at every level. Fifth, preparation is decoction-dominant but integrates dietary therapy in a non-trivial 11% of records. Figure 7 summarises this signature as a conceptual schema; the separate, botanical-drug-specific modern-pharmacology bridge is developed independently in Section 4.2 and should not be read as implying a one-to-one correlation with the five features shown in Figure 7. Read against the historical stratigraphy sketched in the Introduction, this signature is recognizably composite: the formula scaffolds descend from Zhang Zhongjing, the channel-targeted drug attribution from Zhang Yuansu, the tonification-first orientation from Li Dongyuan, and the systematic protection of stomach yin from Zhu Danxi—with Li Shizhen’s contribution being their integration into a single, internally balanced pharmacopoeial system.

**FIGURE 7:**
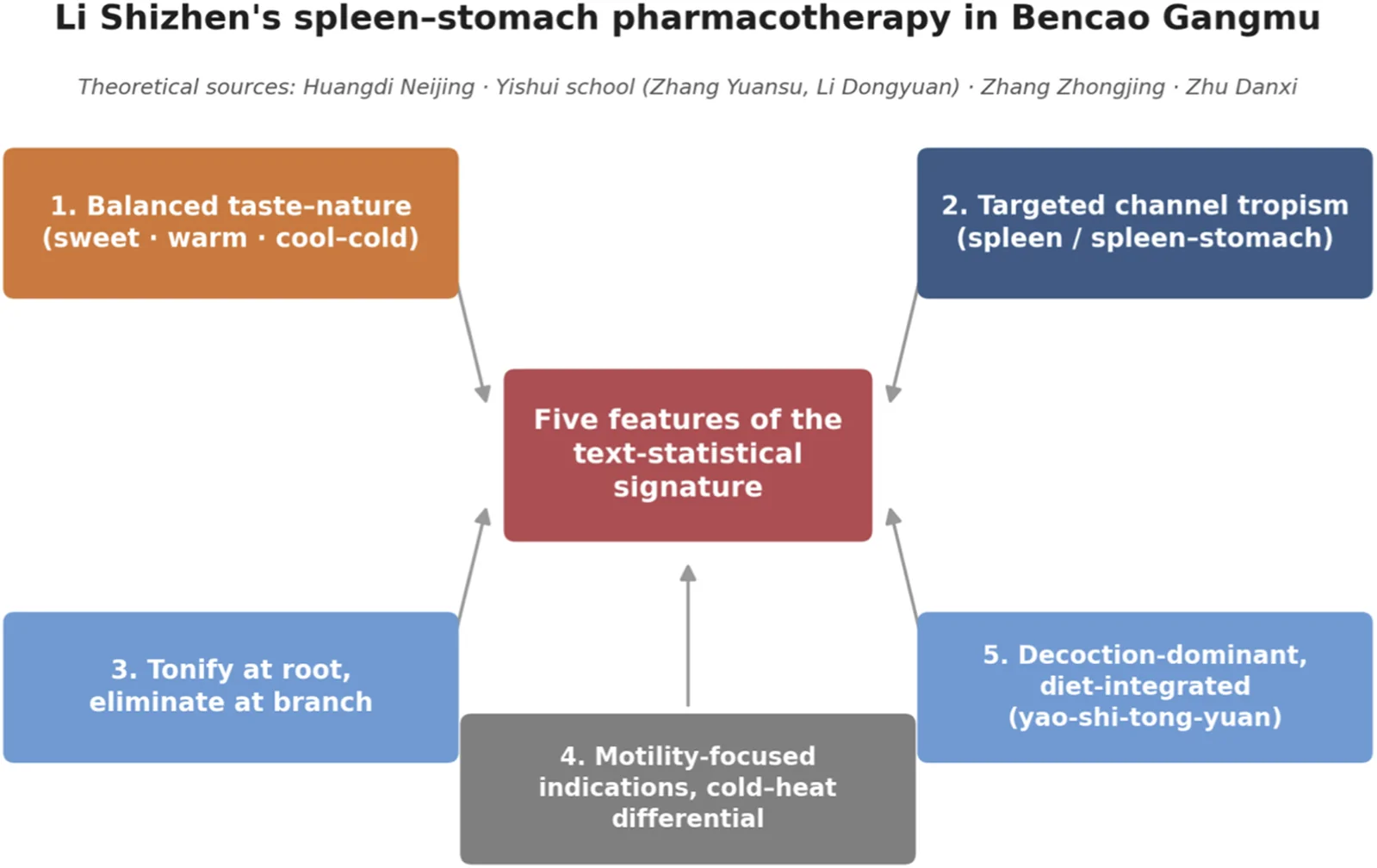
Conceptual schema of the five-feature signature of Li Shizhen’s spleen–stomach pharmacotherapy in Bencao Gangmu. The five peripheral boxes correspond, one-to-one, to the five points developed in Section 4.1 (taste–nature, channel tropism, functional profile, indications, preparation) and converge on a coherent therapeutic logic centred on the dual physiology of the spleen and stomach. Theoretical sources combine Huangdi Neijing, the Yishui school (Zhang Yuansu, Li Dongyuan), Zhang Zhongjing’s classical formulary tradition and Zhu Danxi’s yin-nourishing approach. This schema summarises the text-statistical and doctrinal-historical findings only; it does not depict, and should not be read as asserting, any correlation with the specific metabolites discussed separately in Section 4.2.

### 4.2 A modern-pharmacology bridge for five of the most frequently recorded botanical drugs

The frequency distribution recorded above is not merely of historical interest: each of the most frequent botanical drugs corresponds to a phytochemical class for which modern pharmacological evidence relevant to gastrointestinal pathophysiology is now available. The brief sketch below follows the ConPhyMP standard of identifying the plant by its authenticated binomial, family and organ used, and citing studies in which the plant material was adequately characterized.

#### 4.2.1 *Atractylodes macrocephala* Koidz. (Asteraceae; rhizome; Baizhu)

The rhizome is dominated by sesquiterpene lactones—atractylenolide I, II and III (Hoang et al., 2016) — together with polyacetylenes and *Atractylodes* polysaccharides; recent comprehensive reviews catalogue more than 77 and 120 metabolites respectively and converge on evidence for enhancement of gastrointestinal function, immunomodulation and anti-inflammatory activity (Wang et al., 2026; Luo et al., 2025). Recent experimental models demonstrate that atractylenolide I stimulates intestinal epithelial cell migration and proliferation via a polyamine-mediated Ca^2+^-signalling pathway, accelerating mucosal repair (Song et al., 2017), and separately alleviates diabetic gastroparesis by activating the stem-cell-factor/c-kit pathway (Li et al., 2021). *Atractylodes* polysaccharides exert prebiotic-like effects on the gut microbiota, increasing the abundance of Bacteroidetes and short-chain-fatty-acid producers and modulating host polysaccharide metabolism (Feng et al., 2019; Wang et al., 2014). These findings provide a pharmacological correlate of the historical role of Baizhu as the principal spleen-tonifying drug, and of its dual medicinal–dietary status, which ChP 2025 continues to recognize (Chinese Pharmacopoeia Commission, 2025).

#### 4.2.2 *Zingiber officinale* Roscoe (Zingiberaceae; rhizome; Shengjiang/Ganjiang)

The rhizome contains pungent phenolic alkanones—gingerols (predominantly 6-gingerol), shogaols (formed by dehydration during drying), paradols and zingerone—together with the volatile fraction (Semwal et al., 2015). 6-Gingerol and 6-shogaol accelerate gastric emptying, reduce nausea and vomiting in chemotherapy-induced and pregnancy-related contexts, and exert anti-Helicobacter pylori activity (Nikkhah Bodagh et al., 2019). The transition from fresh ginger (Shengjiang; gingerol-rich, prokinetic-and-antiemetic) to dried ginger (Ganjiang; shogaol-rich, more strongly warming) recorded by Li Shizhen has a clean chemical correlate in the gingerol → shogaol conversion.

#### 4.2.3 *Atractylodes lancea* (Thunb.) DC. (Asteraceae; rhizome; Cangzhu)

The rhizome of *A. lancea* is rich in atractylodin, β-eudesmol and hinesol, with a distinctly different volatile profile from *A. macrocephala*. β-Eudesmol accelerates gastric emptying via cholinergic mechanisms, and atractylodin shows anti-inflammatory activity in experimental colitis (Koonrungsesomboon et al., 2014; Bailly, 2021). Together with *A. macrocephala*, the *Atractylodes* pair instantiates the tonify-and-drain (bu-xiao bing-yong) principle at the level of a single genus.

#### 4.2.4 *Coptis chinensis* Franch. (Ranunculaceae; rhizome; Huanglian)

*Coptis* rhizome is dominated by the isoquinoline alkaloid berberine (4%–9%) together with palmatine, coptisine and epiberberine. Berberine has been the subject of an extensive contemporary literature on glucose homeostasis, lipid metabolism and the gut microbiota (Habtemariam, 2020). In the gastrointestinal context, berberine modulates intestinal barrier function via the AMPK/TJ pathway, attenuates inflammation in experimental colitis, and reshapes gut microbial communities toward an anti-inflammatory profile (Yao et al., 2020). The historical role of Huanglian for stomach-heat syndromes and damp-heat dysentery is consonant with this profile. Remarkably, recent experimental work provides direct support for the cold–heat differential documented in Section 3.6: in high-fat-diet mice modelled with hot *versus* cold syndrome, an aqueous extract of *Coptis chinensis* showed markedly stronger lipid-lowering efficacy and distinct gut-microbiota remodelling in the hot-syndrome model, while its efficacy was blunted under cold syndrome (Yang et al., 2025). This syndrome-dependent pharmacology suggests that the classical restriction of Huanglian to “hot” presentations—explicit in Bencao Gangmu—has a measurable biological correlate.

#### 4.2.5 *Pinellia ternata* (Thunb.) Makino (Araceae; tuber; Banxia)

*Pinellia* tuber must be processed (steamed or soaked in ginger or alum) before use, as the raw material is irritant. Processed Banxia is rich in alkaloids, ephedrine analogues and trace amounts of cyclic peptides; modern studies report anti-emetic activity through central D2 and 5-HT3 receptor modulation (Liu et al., 2019). The classical pairing of Banxia with fresh *Zingiber* (Xiao-Ban-Xia Tang) recorded in Bencao Gangmu is consistent with the synergistic anti-emetic effects of the two phytochemical classes.

### 4.3 Methodological considerations and limitations

Several limitations of this analysis should be acknowledged. First, the analytical unit is the textual entry, not the clinical case; frequency reflects authorial editorial choices rather than the prevalence of clinical practice in the late Ming. Second, the dataset captures co-occurrence rather than formal formula structure; explicit pairing analyses (e.g., via association rules) are beyond the scope of the present paper and are deferred to a companion study. Third, although every plant taxon discussed here has been authenticated under its current binomial, classical names are sometimes ambiguous between species (the *Atractylodes* genus is the leading example); this study has followed the ChP 2025 attribution where it is consistent with the historical description, but residual ambiguity cannot be eliminated. Fourth, the modern-pharmacology bridge in Section 4.2 is illustrative rather than systematic; this study did not attempt a formal scoping review of each botanical drug. In addition, the juxtaposition of the classical text with contemporary phytochemical and pharmacological findings in Section 4.2 is a retrospective, hypothesis-generating device rather than a claim that Li Shizhen or his contemporaries had any access to, or intuition of, the specific metabolites now identified: the historical and phytochemical registers are placed in dialogue without asserting a causal or explanatory link between them. Fifth, classical TCM categories (e.g., spleen deficiency, damp obstruction) do not have one-to-one biomedical equivalents, and any cross-walk is provisional; the glossary in Table 1 offers working renderings, not equivalences. The dataset (Supplementary Data S1) is provided to facilitate future analyses that may address these limitations.

### 4.4 Implications for ethnopharmacology

The methodological contribution of this work is to demonstrate that a foundational pharmacopoeia such as Bencao Gangmu can be converted into a structured, reproducible, query-able ethnopharmacological dataset, and that the resulting frequency distributions are interpretable both in classical TCM terms and against modern pharmacology—an approach complementary to the recent quantitative treatment of the Ayurvedic Pharmacopoeia of India (Yao et al., 2023) and to chemosensation-based analyses of Graeco-Roman materia medica (Leonti et al., 2024). The substantive contribution is a quantitative inventory of Li Shizhen’s spleen–stomach pharmacotherapy that confirms and refines the qualitative consensus in the historical literature: spleen-tonification is dominant but is systematically balanced by stomach-cooling, motility-disorder indications dominate, and dietary integration is non-trivial. The dataset and the methodological template invite extension to other organ systems (e.g., liver–lung axis, kidney pathology) and to other foundational pharmacopoeias (e.g., Shennong Bencao Jing, Tang’s Xinxiu Bencao).

## 5 Conclusion

Bencao Gangmu codifies a coherent spleen–stomach pharmacotherapy whose quantitative signature—sweet–warm dominance with cool counterweight, sharp spleen-and-stomach tropism, tonify-at-root with pathogen-elimination-at-branch, motility-disorder indications, decoction with dietary integration—is recoverable by systematic text-statistical analysis. The five most frequently used botanical drugs converge on phytochemical leads (atractylenolides, gingerols/shogaols, atractylodin/β-eudesmol, berberine, processed-*Pinellia* alkaloids) for which contemporary evidence on gastrointestinal motility, mucosal integrity, gut-microbiota modulation and anti-inflammatory activity is now available. The dataset is offered as a hypothesis-generating ethnopharmacological resource and as a reproducible methodological template for further text-statistical studies of historical materia medica.

## Data Availability

The datasets presented in this study can be found in online repositories. The names of the repository/repositories and accession number(s) can be found below: github https://github.com/arielsun1990/Supplementary-Data.
