# Supplementary material for: Pharmacotherapy of the spleen and stomach in Li Shizhen’s Bencao Gangmu (Compendium of materia medica, 1596): a text-statistical analysis of 994 entries and a bridge to modern pharmacology
Source: Front Pharmacol. 2026 Sep 16;17:1889778. doi: 10.3389/fphar.2026.1889778 (PMC13624087; doi:10.3389/fphar.2026.1889778)
Supplement: Supplementary file 1 [file Supplementaryfile1.docx]

**Supplementary Table S1. Authenticated plant identities for the herbs discussed in Sun et al.**

*Plant identities follow World Flora Online (https://www.worldfloraonline.org/) and Plants of the World Online (POWO; https://powo.science.kew.org/). Family attribution follows APG IV. Pharmacopoeia reference is to the Pharmacopoeia of the People's Republic of China, 2025 edition (Volume I).*

| **Pinyin name** | **Latin binomial (authenticated)** | **Family** | **Plant part used** | **Processing** | **ChP 2025** |
| --- | --- | --- | --- | --- | --- |
| Baizhu | *Atractylodes macrocephala Koidz.* | Asteraceae | Rhizome | Raw / bran-fried / earth-fried | Yes |
| Shengjiang | *Zingiber officinale Roscoe* | Zingiberaceae | Fresh rhizome | Raw | Yes |
| Cangzhu | *Atractylodes lancea (Thunb.) DC.* | Asteraceae | Rhizome | Raw / bran-fried | Yes |
| Banxia | *Pinellia ternata (Thunb.) Makino* | Araceae | Tuber | Processed (alum / ginger / lime) | Yes |
| Dingxiang | *Syzygium aromaticum (L.) Merr. & L.M.Perry* | Myrtaceae | Flower bud | Dried | Yes |
| Huanglian | *Coptis chinensis Franch.* | Ranunculaceae | Rhizome | Raw / wine-fried / ginger-fried | Yes |
| Wuzhuyu | *Tetradium ruticarpum (A.Juss.) T.G.Hartley (syn. Evodia rutaecarpa)* | Rutaceae | Unripe fruit | Liquorice-processed | Yes |
| Houpo | *Magnolia officinalis Rehder & E.H.Wilson* | Magnoliaceae | Stem bark / root bark | Ginger-fried | Yes |
| Fuzi | *Aconitum carmichaelii Debx.* | Ranunculaceae | Daughter root | Processed (heishunpian, baifupian) | Yes |
| Muxiang | *Aucklandia costus Falc. (syn. Saussurea costus)* | Asteraceae | Root | Raw / wei-roasted | Yes |
| Huangqin | *Scutellaria baicalensis Georgi* | Lamiaceae | Root | Raw / wine-fried | Yes |
| Hujiao | *Piper nigrum L.* | Piperaceae | Unripe / ripe fruit | Raw | Yes |
| Ganjiang | *Zingiber officinale Roscoe* | Zingiberaceae | Dried rhizome | Dried | Yes |
| Zhishi | *Citrus × aurantium L.* | Rutaceae | Immature fruit (dried, small) | Bran-fried | Yes |
| Shengma | *Cimicifuga heracleifolia Kom.* | Ranunculaceae | Rhizome | Raw | Yes |
| Shanyao | *Dioscorea polystachya Turcz.* | Dioscoreaceae | Rhizome | Raw / bran-fried | Yes |
| Lianzi | *Nelumbo nucifera Gaertn.* | Nelumbonaceae | Mature seed | Raw | Yes |
| Gaolianjiang | *Alpinia officinarum Hance* | Zingiberaceae | Rhizome | Raw | Yes |
| Shigao | *Gypsum fibrosum (mineral, CaSO4·2H2O)* | — | Mineral; not a botanical | Raw / calcined | Yes |

*ChP 2025 = Pharmacopoeia of the People's Republic of China, 2025 edition, Volume I. “Yes” indicates the drug is retained as a standard monograph; page-specific citations are omitted pending verification against the finalized 2025 print edition.*
